# Mechano-Physical Properties and Microstructure of Carbon Nanotube Reinforced Cement Paste after Thermal Load

**DOI:** 10.3390/nano7090267

**Published:** 2017-09-11

**Authors:** Maciej Szeląg

**Affiliations:** Faculty of Civil Engineering and Architecture, Lublin University of Technology, 40 Nadbystrzycka Str., 20-618 Lublin, Poland; maciej.szelag@pollub.pl; Tel.: +48-81-538-4428

**Keywords:** carbon nanotubes, cement paste, elevated temperature, sodium dodecyl sulfate (SDS), water dispersion

## Abstract

The article presents the results obtained in the course of a study on the use of carbon nanotubes (CNTs) for the modification of a cement matrix. Carbon nanotubes were introduced into a cement paste in the form of an aqueous dispersion in the presence of a surfactant (SDS—sodium dodecyl sulfate), which was sonicated. The selected physical and mechanical parameters were examined, and the correlations between these parameters were determined. An analysis of the local microstructure of the modified cement pastes has been carried out using scanning electron microscope (SEM) and X-ray microanalysis (EDS). In addition, the effect of carbon nanotubes on the change in characteristics of the cementitious material exposed to the sudden, short-term thermal load, was determined. The obtained material was characterized by a much lower density than a traditional cement matrix because the phenomenon of foaming occurred. The material was also characterized by reduced durability, higher shrinkage, and higher resistance to the effect of elevated temperature. Further research on the carbon nanotube reinforced cement paste, with SDS, may contribute to the development of a modified cement binder for the production of a lightweight or an aerated concrete.

## 1. Introduction

Cement composites are one of the most widely used construction materials in the world. The reasons for this are, primarily, technological ease of production, availability of raw materials necessary for production, low cost of production, high durability over many decades of operation, the possibility of making any shape, recyclability, and much more [1].

Increasing demands on the durability of cementitious materials, the emergence of new and much more complex constructions, and the aggressive environment, require researchers to seek new methods and ways to improve the performance of concrete and the cement matrix itself. The necessity of examining the characteristics of the cement paste is highly justified, because it is the basic ingredient of mortars and concretes. The cement matrix defines, primarily, the technical and functional characteristics of the cementitious composite [1,2,3].

In order to improve the properties of the cement matrix, a number of additives and admixtures are used, including e.g., pozzolanic additives (microsilica, fly ash, metakaolinite, zeolites, etc.) [1,4]. A separate group of modifications use scattered reinforcement in the form of various types of fibers, e.g., steel, polypropylene, basalt, glass, aramid, natural, etc. [5,6,7,8]. In recent years, research into the use of carbon nanotubes (CNTs) as a nano-reinforcement of cement matrix has been conducted in many countries [9,10,11,12,13].

Carbon nanotubes, due to their particular mechanical, thermal, and electrical characteristics, have revolutionized the electronics industry and have become the basis of nanotechnology as a discipline of technology and science [14]. CNTs are rolled up graphene plates. Such a structure can have a fully enclosed surface, because nanotubes are ended at one or both ends by the semicircular canisters. CNTs’ characteristic feature are their large ratio of length to diameter. They can be single-, double-, and multi-wall [15]. The methods of CNT production are very complex, which makes it now an expensive material. The common feature of the CNT manufacturing process is the slow condensation of a hot steam of carbon atoms. The obtained nanotubes take two forms: the first is deposit made of tangled, amorphous and randomly mixed nanotubes, the second form is a row of parallel nanotubes on a catalyst supported substrate [16,17].

The CNT’s morphology is unique. This material is characterized by a very large surface area, new and variable electronic properties, a very high Young’s modulus (about 2 TPa), very strong bonds between carbon atoms that give unmatched durability, high tensile strength (about 50 GPa) and bending strength, and very good conductivity of heat and electricity. Their diameter usually oscillates in the range from 1 to 100 nm, and length, from 10 nm to 10^−2^ m [16,18,19].

For many years, research has been ongoing on the use of CNTs in medicine and electronics. An exceptionally new approach is the use of CNTs in concrete technology as a nano-reinforcement [20], in which a small addition can improve the mechanical properties of a cementitious composite. In some studies [21,22], it has been shown that the use of multi-wall carbon nanotubes positively affects the nano- and micro-mechanical properties of a cement paste. In addition, the cracking resistance of the cement matrix, and the local stiffness of the calcium-silicate-hydrate (CSH) phase has been increased. It has also been shown that the porosity of cement matrix has been reduced. The studies [23] confirmed a decrease of the local porosity after the application of multi-wall carbon nanotubes. An increase in the compressive strength of cementitious composites was also observed. With the use of the scanning electron microscope (SEM) it was noted that CNTs create bridges between nano- and micro-cracks in the binder. This resulted in increased tensile strength and limiting further the cracks’ propagation—the same conclusions were made by the American scientists [24]. Based on the above-mentioned studies, CNTs are dosed in the amount of 0.04% to 0.5% by weight of cement.

The biggest problem in the use of CNTs as an additive to cementitious composites is their uneven dispersion and low adhesion to cement paste. Applying the CNTs alone to the paste does not produce any effect, as they tend to agglomerate, due to their large surface area. Most often, CNTs are used as an aqueous suspension in the presence of a surfactant that has been previously sonicated, in order to obtain a high CNT dispersion. For this purpose, the supercritical-fluid chromatography (SFC) techniques were used [21,22]. As a surfactant among others, H_2_SO_4_, HNO_3_ [23,25], and isopropanol [24] were used. A stable dispersion of CNTs is also obtained in the presence of SDS (sodium dodecyl sulfate) [26,27,28,29,30,31], NaDDBS (sodium dodecyl benzene sulfonate), and DMAc (di-methyl acetamide) [26]. A very high degree of CNT dispersion and stable aqueous solution (stable for a few months) is obtained in the presence of SDS, which was investigated by UV spectroscopy and transmission electron microscopy (TEM) [27]. Some studies on the use of the CNT dispersion with SDS [32,33,34], NaDDBS [35], and DMAc for cementitious composites, were conducted, but this aspect is not yet well researched.

The article presents research on the use of CNTs as a nano-reinforcement of cement matrix. Selected physical (apparent density, linear shrinkage) and mechanical parameters (compressive strength and bending tensile strength) of the CNT modified pastes were examined, and the correlations between these parameters were calculated. CNTs with SDS (as a surfactant) were used as an aqueous dispersion, which was sonicated. An analysis of the microstructure of cement matrix using scanning electron microscope (SEM) and X-ray microanalysis (EDS) was also performed.

In addition, the CNT modified cementitious material was subjected to a sudden, short-term thermal load (thermal shock), and the selected physical and mechanical properties of the material were redefined. So far, no studies have been published in the literature, in which the effect of CNT addition on the modification of the cement matrix performance under thermal shock was determined.

## 2. Materials and Methods

### 2.1. Manufacturing of the Modified Cement Pastes

This study was conducted on four series of cement paste specimens. For each series, samples were made with three different water/cement ratios (*w*/*c*) equal to 0.4, 0.5, and 0.6. Two series were a classical, unmodified cement matrix—a combination of water and cement. In the other two series, CNT were added in the amount of 0.1% by weight of cement. The following cement paste series were made for this study:

- C42—consisting of CEM I 42.5R and water,
- C42CNT—consisting of CEM I 42.5R, 0.1% CNT, and water,
- C52—consisting of CEM I 52.5R and water,
- C52CNT—consisting of CEM I 52.5R, 0.1% CNT, and water.

Two Portland cements—CEM I 42.5R and CEM I 52.5R (Cemex, Chełm, Poland) were used for this study. CEM I 42.5R is guaranteed to reach the minimum strength of 42.5 MPa after 28 days; CEM I 52.5R—52.5 MPa after 28 days. Cements have a very similar chemical composition, but differ significantly in a specific surface area, as shown in Table 1. The similar oxide composition of the cements directly translates to a similar mineral composition (calculated by Bogue’s formula [36]).

**Figure 1:**
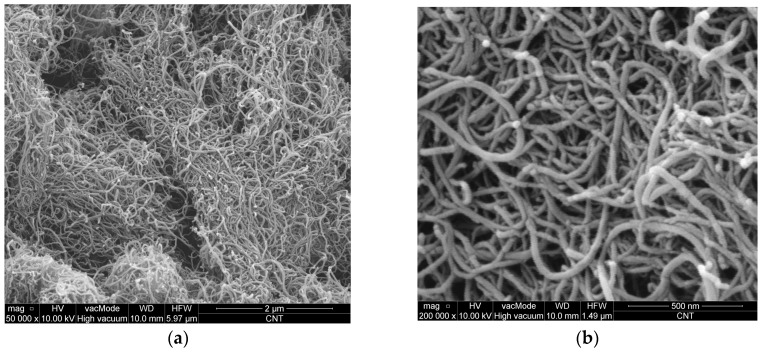
SEM images of carbon nanotubes (CNTs) used: (**a**) magnification 50,000×; (**b**) magnification 200,000×.

As a nano-reinforcement, multi-wall carbon nanotubes were used—Nanocyl™ NC7000 (Nanocyl, Sembreville, Belgium). They were dosed in the amount of 0.1% by weight of cement. Their average diameter is 9.5 nm, average length—1.5 μm, specific surface area—250–300 m^2^/g, tensile strength—about 60 GPa, Young’s modulus—1 TPa, carbon purity—90%, and metal oxide—10%. CNTs used in this study are shown in Figure 1, which was taken by means of a SEM.

As a surfactant, SDS (sodium dodecyl sulfate—C_12_H_25_OSO_2_ONa—MERCK Milipore, Billerica, MA, USA) was used, in order to prepare the CNT dispersion. SDS used is a white powder, with a specific density of 1.1 g/cm^3^, bulk density of 0.49–0.56 g/cm^3^, and pH of 6–9.

### 2.2. Preparation of Mixture and Specimens

The paste was made by mechanically mixing cement with water. In the recipes that contained CNT (C42CNT and C52CNT), initially their aqueous dispersion was made, and then it was mixed with cement. The CNT dispersion was made with a surfactant (SDS), in a 1:5 weight ratio (CNT/SDS).

**Figure 2:**
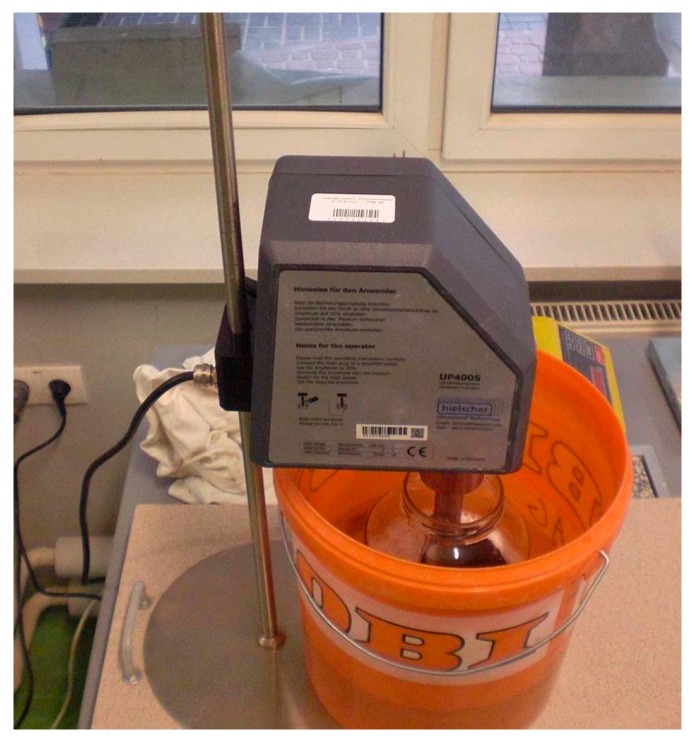
Laboratory stand for the production of the CNT suspension.

**Figure 3:**
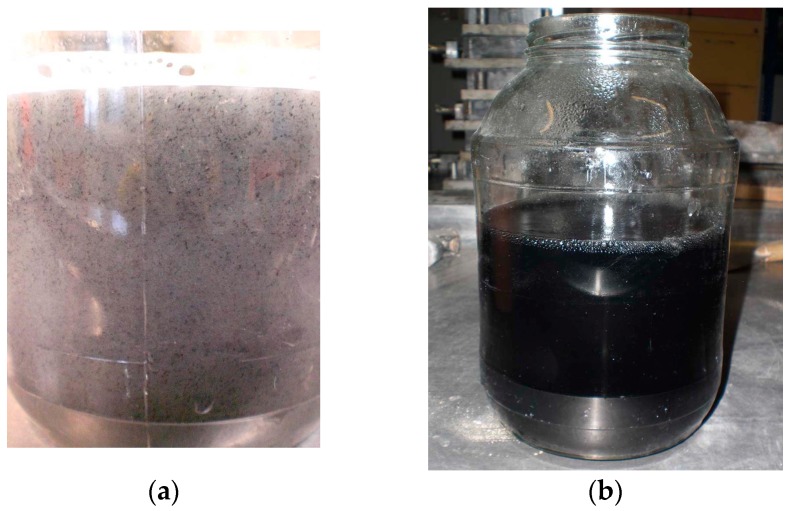
The CNT water suspension: (**a**) before sonication—visible clusters of CNT in the form of “lumps”; (**b**) after sonication—uniform color of the suspension, which indirectly correspond to a uniform CNT dispersion.

The aqueous solution was sonicated using an UP400S ultrasonic homogenizer (Hielscher Ultrasonics Gmbh, Teltow, Germany). The H22 sonotrode (Hielscher Ultrasonics Gmbh, Teltow, Germany) with a tip diameter of 22 mm, a maximum amplitude of 100 μm, and a sound pressure density of 85 W/cm^2^, was used. The homogenizer has a constant operating frequency equal to 24 kHz. During sonication, the homogenizer was set to continuous mode, with the output power set at the maximum level, which in conjunction with the H22 sonotrode, gave 300 W of power. The CNT dispersion was sonicated in a glass jar for 30 min, which was inserted into a bucket with cold water to expedite the heat transfer generated during ultrasonic mixing. The laboratory stand at which sonication was performed is shown in Figure 2. Conversely, Figure 3 shows the CNT aqueous dispersion before and after sonication.

All samples were made into bars for proper paste and mortar testing, with dimensions of 40 × 40 × 160 mm^3^. The mixture was laid into molds in two layers, and sequentially compacted using a standardized vibrator, according to PN-EN 196-1 [37]. Then all samples were stored for 28 days in air-dry conditions with an average relative humidity of 50%.

### 2.3. Thermal Load

After 28 days of maturation, the samples were subjected to a short-term thermal load. The furnace was heated to 250 °C, then the samples were put into it for 4 h. For this purpose, the SLW-400 ECO furnace (POL-EKO-APARATURA sp.j., Wodzisław Śląski, Poland) with forced air circulation was used; the furnace operates in the temperature range from +5 to +250 °C. After the samples were heated, they were removed from the furnace, and the cooling was effected by a natural temperature drop at room temperature (about 20 °C). Placing the samples in the preheated furnace can be termed as the thermal shock.

The parameters of the temperature load were selected on the basis of tests carried out at different temperatures and at different load duration, within the framework of previous authors’ studies [2,38,39,40,41]. In the studies, the surface structure of the thermal cracks of the modified cement pastes was analyzed, using computer image analysis.

### 2.4. Methods

#### 2.4.1. Determination of Physical Properties

Determination of an apparent density (*D*) of the hardened cement paste was carried out in accordance with EN 12390-7 [42]. Due to the regular shape (rectangular), the specimens have been measured by a caliper in any of the three directions, and then weighed on a laboratory scale. The apparent density has been calculated on the basis of these data. The physical feature was measured for samples after 28 days of maturing (*D*_(*R*)_), as well as on samples after the temperature load, and cooled down to the room temperature (*D*_(*T*)_). Results presented in this study are an arithmetic means of 6 samples.

The linear shrinkage (*S*) determination was performed on a Graf–Kaufman device (ToRoPol Sp. z o.o., Warsaw, Poland). This method requires the installation of special metal pins in the specimens’ faces. They were fixed at the time of placing the cement paste in molds specially designed for this. Shrinkage was determined after 3 (*S*_3_), 7 (*S*_7_), 14 (*S*_14_), 21 (*S*_21_), and 28 (*S*_28(*R*)_) days of maturation, and on samples subjected to the temperature load, and cooled to the room temperature (*S*_28(*T*)_). The first measurement was made immediately after the molds were removed (24 h after the paste was placed). Results for each series, and the *w*/*c* ratio, are the arithmetic means of the 6 measurements.

#### 2.4.2. Determination of Mechanical Properties

The bending tensile strength test (*f_ct_*_,*f*_) using three-point bending according to EN 12390-5 [43], was performed. The parameter was calculated using the modulus of rupture (MOR) formula. The strength was examined for samples after 28 days of maturing (*f_ct_*_,*f*(*R*)_), as well as on samples after the temperature load, and cooled down to the room temperature (*f_ct_*_,*f*(*T*)_). Results presented in this study are the arithmetic means of 6 samples. Samples were loaded perpendicular to the direction of molding. The specimens’ halves were used for the compressive strength test. The bending tensile strength test was conducted using the MTS 810 press with the 10 kN head.

The compressive strength test (*f_c_*) according to EN 12390-3 [44] was performed. As in the case of the bending tensile strength, the compressive strength test was carried out on the samples after 28 days of maturation (*f_c_*_(*R*)_), and on the heat-treated samples (*f_c_*_(*T*)_). Results presented are the arithmetic means of the 12 measurements. The test was carried out using the CONTROLS Advantest 9 press (CONTROLS, Milan, Italy), with an arm with a maximum pressure up to 250 kN, and the compression strength device to test 40 × 40 × 40 mm^3^ specimens. Samples were compressed in a perpendicular direction to the molding. 

#### 2.4.3. Microstructure Analysis

Analysis of a local microstructure of the modified cement pastes was performed on the basis of images obtained from the scanning electron microscope (SEM)—Quanta Feg 250 (FEI, Hillsboro, OR, USA). The analysis was carried out after 28 days of maturation on the heat-treated samples (250 °C for 4 h), after cooling the samples to room temperature. Samples were sputtered by carbon before analysis. Images were taken in the high vacuum mode with magnifications from 100× to 200,000×.

On the same samples, the EDS (Energy Dispersive X-ray Spectroscopy, FEI, Hillsboro, OR, USA) analysis was performed in order to identify the chemical composition of material. The results are presented in terms of weight content of oxide equivalents. Series C52 and C52CNT with *w*/*c* = 0.4 were analyzed.

## 3. Results

### 3.1. Physical Properties of the Modified Cement Pastes

**Figure 4:**
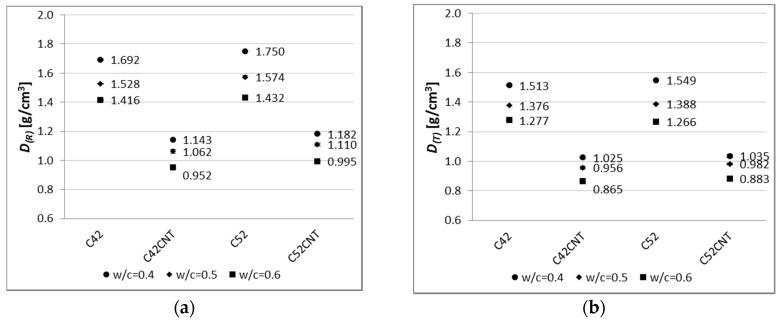
Apparent density of modified cement pastes (error bars mean standard deviations of apparent density; due to the very small values, the error bars are invisible) for (**a**) reference samples; (**b**) samples after thermal load.

The apparent density of all analyzed series is presented in Figure 4. The standard deviation reached very small values; 0.002–0.012 for the reference samples, and 0.001–0.010 for the heat-treated samples.

**Figure 5:**
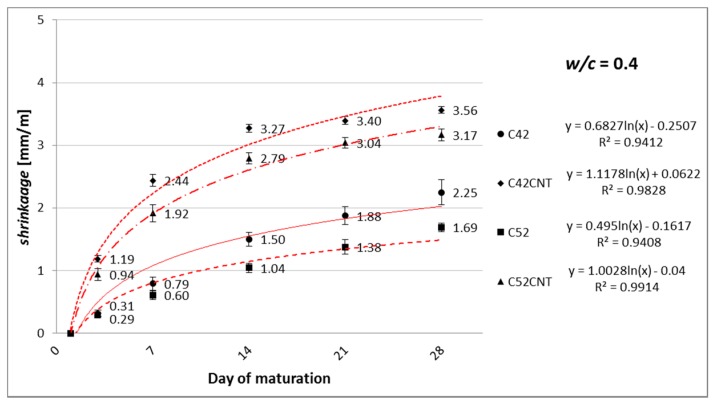
Increase in linear shrinkage of modified cement paste with *w*/*c* = 0.4, as a function of maturation time.

**Figure 6:**
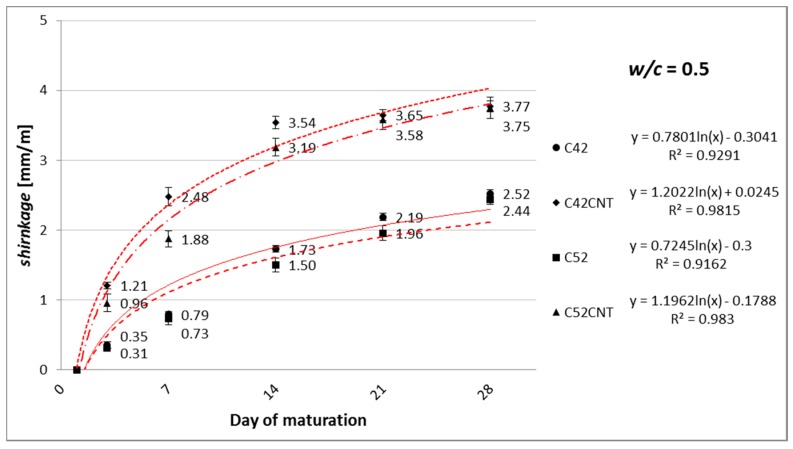
Increase in linear shrinkage of modified cement paste with *w*/*c* = 0.5, as a function of maturation time.

**Figure 7:**
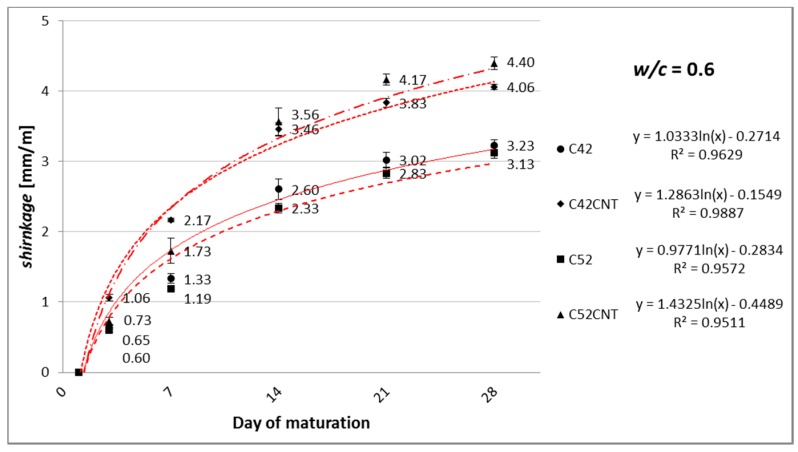
Increase in linear shrinkage of modified cement paste with *w*/*c* = 0.6, as a function of maturation time.

Figure 5, Figure 6 and Figure 7 show the linear shrinkage after 3, 7, 14, 21, and 28 days of maturation of cement paste, respectively, for *w*/*c* = 0.4, 0.5, and 0.6.

Table 2 shows the linear shrinkage values after thermal load, the increase in shrinkage, and decrease in apparent density after thermal shock.

### 3.2. Mechanical Properties of the Material

**Figure 8:**
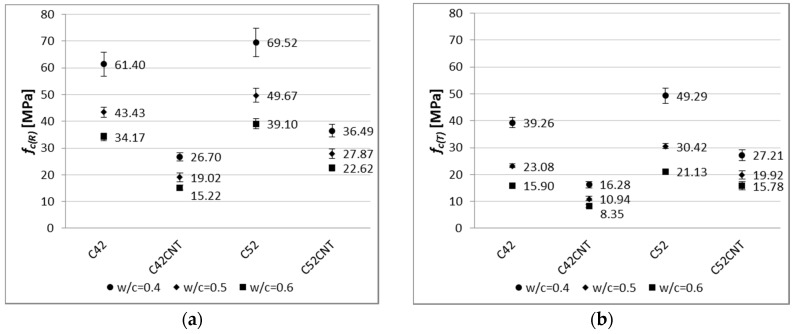
Compressive strength of modified cement pastes (error bars mean standard deviations of compressive strength) for (**a**) reference samples; (**b**) samples after thermal load.

Figure 8 shows the compressive strength of all analyzed series of cement paste.

**Figure 9:**
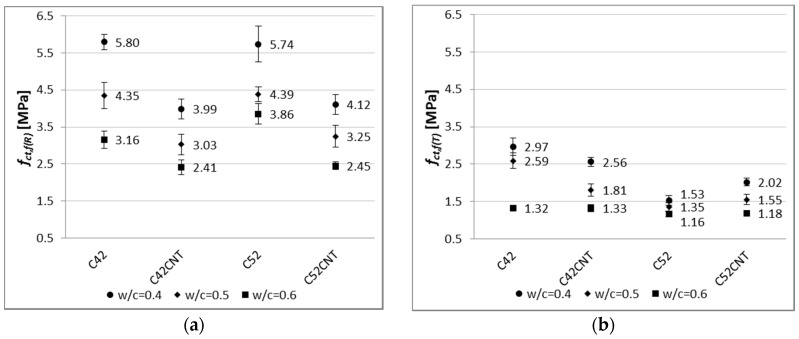
Flexural strength of modified cement pastes (error bars mean standard deviations of flexural strength) for (**a**) reference samples; (**b**) samples after thermal load.

Figure 9 shows the flexural strength of all tested cement paste series.

Table 3 shows the decline in the bending tensile and compressive strength after the thermal load.

### 3.3. SEM and EDS Analysis of the Material

**Figure 10:**
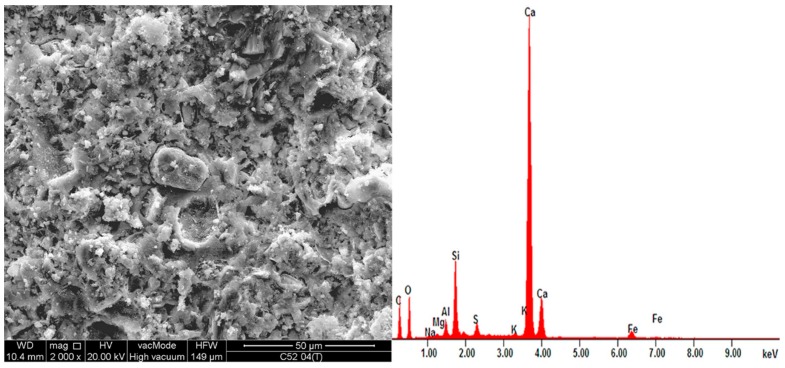
EDS spectra of series C52 with *w*/*c* = 0.4 together with the SEM image of the analyzed area.

**Figure 11:**
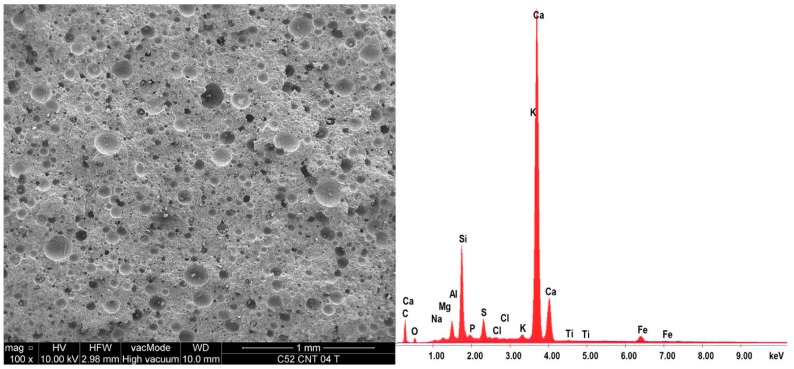
EDS spectra of series C52CNT with *w*/*c* = 0.4 together with the SEM image of the analyzed area.

Figure 10 and Figure 11 show sample SEM images for series C52 and C52CNT, and EDS spectra for these areas. Table 4 shows the oxide composition of the two samples with *w*/*c* = 0.4. More SEM images are shown and discussed in detail in the Discussion section.

## 4. Discussion

### 4.1. Physical Properties of the Modified Cement Pastes

#### 4.1.1. Apparent Density

Apparent density is one of the basic physical parameters of building materials. One of the variables determining the density of the cement paste is the *w*/*c* ratio. Based on Figure 4, it has been noted that as the *w*/*c* increases, the apparent density decreases. Thus, samples with *w*/*c* = 0.5 and 0.4 reached greater *D*_(*R*)_ values by an average of 10% and 20% respectively, than samples with *w*/*c* = 0.6. Identical differences were observed between the heat-treated samples. Cement has a density of about three times higher than water. Due to the reduction in the amount of cement in the material’s volume (increase in *w*/*c*), the amount of water is increased, so the cementitious material has a lower resultant density. The apparent density is also dependent on the volume of the capillary pores that are formed during the cement hydration process. According to Power’s law, under identical curing conditions, the capillary pores’ volume is shaped by the *w*/*c* ratio—the higher *w*/*c*, the higher the capillary pores volume [45]. Water, when evaporating, leaves more empty pores if a higher *w*/*c* ratio is used, which results in a lower apparent density of cementitious material.

The cement pastes made of CEM I 52.5R (C52, C52CNT) were characterized by 3% and 1% higher *D*_(*R*)_ and *D*_(*T*)_ values than the series C42 and C42CNT. The impact of the cement’s class is negligible on the density of the cement matrix. However, the cement of higher class (CEM I 52.5R) has a larger surface area, and therefore, its grains are smaller than the CEM I 42.5R grains. As the size of the grains decreases, according to the Power’s law mentioned above, the distance between them is also smaller, which results in smaller capillary pore volume. In such a situation, the volume of the hydration products is larger, resulting in a slight increase in apparent density. 

The use of the aqueous CNT dispersion was the main factor causing the change of apparent density. Series C42CNT and C52CNT were characterized by a lower apparent density, by an average of 31%, than series C42 and C52. After the thermal load, the above difference was the same.

**Figure 12:**
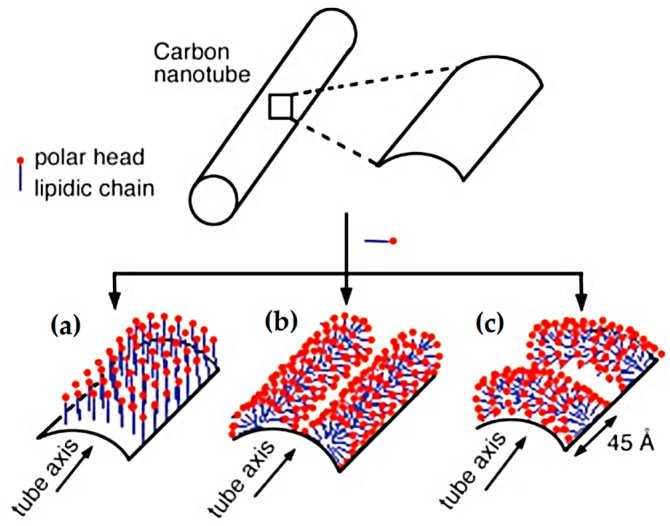
Different possible organizations of the SDS molecules on the surface of a CNT: (**a**) perpendicular adsorption; (**b**) half-cylinders oriented parallel to the tube axis; (**c**) half-cylinders oriented perpendicular to the tube axis [47]. Reproduced with permission of [47]. Copyright AAAS, 2003.

**Figure 13:**
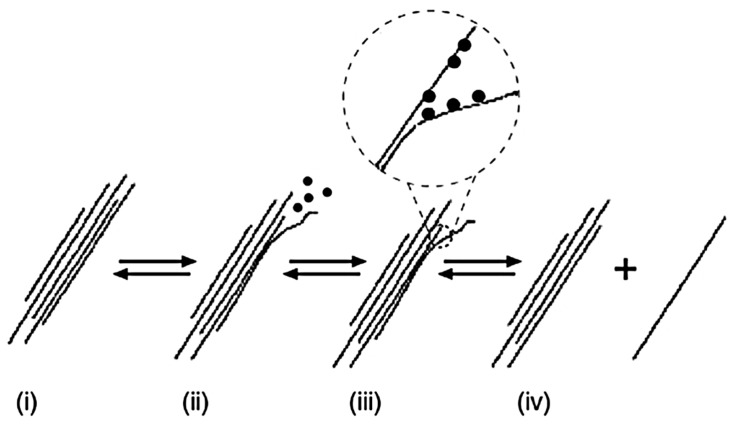
Mechanism of nanotube isolation from bundle obtained by sonication and surfactant stabilization: (**i**) nanotubes bundle; (**ii**) providing high local shear by sonication; (**iii**) surfactant adsorption; (**iv**) separation of the individual nanotube from the bundle [48]. Reproduced with permission of [48]. Copyright American Scientific Publishers, 2003.

The use of the CNT dispersion with SDS resulted in the formation of air pores during the mixing of cement paste, which were enclosed in the material’s volume. A similar result was obtained by Sobolkina et al. [46]. SDS, as an anionic surfactant, is commonly used as a detergent component, and exhibits characteristics specific to blowing agents—gaseous and foaming substances. As a surfactant, SDS adsorbs on the surface of the CNTs during sonication, and reduces the surface tension in contact with the aqueous phase. Possible ways of SDS adsorption on the CNT’s surface have been investigated by Richard et al. [47], as schematically shown in Figure 12. The mechanism of nanotube isolation from a bundle (Figure 13i), with the combined assistance of sonication and surfactant adsorption, was proposed by Strano et al. [48]. The role of ultrasonic treatment is likely to provide high local shear, particularly to the nanotube bundle end (ii); Once spaces or gaps at the bundle ends are formed, they are propagated by surfactant adsorption (iii); ultimately separating the individual nanotubes from the bundle (iv).

The use of the CNT dispersion with SDS resulted in the formation of air pores during the mixing of cement paste, which were enclosed in the material’s volume. A similar result was obtained by Sobolkina et al. [46]. SDS, as an anionic surfactant, is commonly used as a detergent component, and exhibits characteristics specific to blowing agents—gaseous and foaming substances. As a surfactant, SDS adsorbs on the surface of the CNTs during sonication, and reduces the surface tension in contact with the aqueous phase. Possible ways of SDS adsorption on the CNT’s surface have been investigated by Richard et al. [47], as schematically shown in Figure 12. The mechanism of nanotube isolation from a bundle (Figure 13i), with the combined assistance of sonication and surfactant adsorption, was proposed by Strano et al. [48]. The role of ultrasonic treatment is likely to provide high local shear, particularly to the nanotube bundle end (ii); Once spaces or gaps at the bundle ends are formed, they are propagated by surfactant adsorption (iii); ultimately separating the individual nanotubes from the bundle (iv).

As a result of the process described above, there is a formation of the CNT suspension with a high degree of dispersion in the volume of the dispersion medium—water in this case. The solution has a higher viscosity compared to water, so when cement is added, the resulting cement paste may undergo foaming in the course of mixing. Thus, the cementitious material is characterized by a much lower density.

The decrease in apparent density after thermal shock (Table 2) was similar for all samples. It has been noted that a slight difference exists in the criterion of cement used. The apparent density decrease for C42 and C42CNT samples was in the range of 9.2–10.6%, while for C52 and C52CNT—11.3–12.4%. Drop in apparent density after heating is due to the evaporation of free water from the material’s volume. No CNT additive was found to affect this characteristic.

#### 4.1.2. Linear Shrinkage

As the cement content increases (and decreases the amount of water) in the material’s volume (decrease in *w*/*c*), cement matrix tends to experience smaller linear shrinkage. This phenomenon has been confirmed in this study, and this is shown in Figure 5, Figure 6 and Figure 7. This relationship is particularly evident with the increase in the sample’s maturation time. Shrinkage after 28 days, for samples with *w*/*c* = 0.5, was lower by an average of 16%, and 28% for samples with *w*/*c* = 0.4, compared to *w*/*c* = 0.6 specimens. Due to the maturation of samples in the air-dry conditions, with an average relative humidity of 50%, the main factor shaping shrinkage (especially after 14–28 days of maturation) is the drying shrinkage associated with the evaporation of free water contained in samples.

It has been observed that the growth rate of shrinkage decreases during the maturation time. Within the first 7 days, unmodified cement pastes (C42, C52) achieved 36% of the shrinkage measured after 28 days. On the other hand, the CNT samples—56%. Increased porosity of the CNT pastes allows greater matrix deformation due to the removal of free water from the material. This state translates into an intense increase in shrinkage, especially in the initial stage of maturation. In addition, shrinkage from autogenic processes is the greatest in the early phase of the cement binding.

There was no significant effect of the cement’s class on the results obtained after 28 days (average difference was only 4%). In this respect, the chemical composition of the cement is most affected. However, both cements used in this study are chemically very similar (Table 1).

Adding CNTs to cement paste, in the form of the aqueous dispersion with SDS, had a negative effect on shrinkage. After 3 days of maturation, the series C42CNT and C52CNT achieved higher values of the physical parameter by an average of 140%, compared to the series C42 and C52. The relative increase in shrinkage diminished over time, and after 28 days of maturation, the CNT samples reached a higher shrinkage of 49% than the classical cement paste. It is worth noting that despite the rapid increase in shrinkage in the first two weeks of maturation, the absolute increase in shrinkage, between 14 and 28 days for the series C42CNT and C52CNT, was significantly lower than the unmodified cement paste.

By analyzing the increase in shrinkage (Δ*S*) after thermal shock (Table 2), it was observed that this value increases as the *w*/*c* ratio increases. However, there was no significant influence of the cement’s class on this parameter. The shrinkage size of cement paste after thermal shock is the result of two factors. At the stage of heating, cement paste expands, due to the thermal expansion. At the same time, there is strong water evaporation from the material’s structure, which results in shrinkage that increases with an increase in water content in the sample’s volume (increase in *w*/*c*). From the large and interconnected capillaries, water can easily evaporate.

Significant increase in shrinkage was observed for samples with CNTs (average 67% higher than the C42 and C52 samples), but the percentage increase was similar to the unmodified specimens, and ranged from 135% to 174%, in comparison with the values obtained before the thermal load. This is due to the above discussed relationship with increased porosity of the cementitious material.

**Figure 14:**
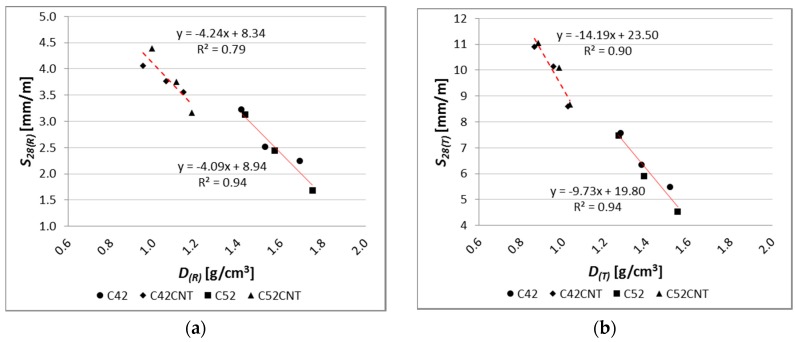
Relationship between linear shrinkage and apparent density of tested materials for (**a**) reference samples; (**b**) samples after thermal load.

A close relation between shrinkage and apparent density of the examined pastes was observed (Figure 14). Empirical data were considered in the CNT application criterion, and the proposed linear regression models, to a large degree, reflect these data. The coefficient of determination of the linear regression equation (*R*^2^) for the series C42 and C52, has a very high value, equal to 0.94, both before and after the exposure to the elevated temperature. For the CNT modified pastes (C42CNT and C52CNT), the *R*^2^ value is equal to 0.79 and 0.90, respectively, for the reference and heat-treated samples.

As the apparent density of cement paste is a function of its porosity, the above relationship confirms previous considerations that one of the major factors influencing the shrinkage of the cement matrix is its porosity.

### 4.2. Mechanical Properties of the Examined Material

#### 4.2.1. Compressive Strength

The characteristic for cement composites is that the smaller *w*/*c* ratio, the higher values of mechanical parameters. This has been confirmed in this study, for both the reference and post-temperature samples (Figure 8). The reference samples with *w*/*c* = 0.5 and 0.4 achieved higher *f_c_*_(*R*)_ values by an average of 26% and 75%, respectively, than samples with *w*/*c* = 0.6. Considering this dependence after thermal shock, these differences are even greater—38% and 116%, respectively.

By analyzing the results of compressive strength in the cement used criterion, it was found that the higher values (both before and after the influence of temperature) were obtained by samples made of the higher class cement, i.e., CEM I 52.5R (series C52 and C52CNT). The effect of the cement’s class is more visible for the CNT material, as the C52CNT samples have reached higher *f_c_*_(*R*)_ and *f_c_*_(*T*)_ values by an average of 43% and 77%, respectively, than the series C42CNT. On the other hand, the above relationship for the classical cement paste (C52 compared to C42) is much lower—14% and 29%, respectively.

The experience of other researchers indicates that the introduction of additional porosity into the material’s volume is beneficial, in terms of some physical properties, e.g., density, heat transfer coefficient, capillary rise. However, the presence of extra pores in the material, which cause discontinuity of the structure, usually results in a deterioration in mechanical properties [49,50,51]. It is similar in the case of the cement pastes tested, in which series C42CNT reached 56% and 55% of compressive strength of the series C42, for *f_c_*_(*R*)_ and *f_c_*_(*T*)_, respectively. For the series in which CEM I 52.5R was used (C52CNT compared to C52), these differences are equal to 45% and 38%, respectively. The negative strength effect is reduced with the use of the higher class cement. This is a valuable guideline for practical use of the CNT cement matrix with SDS.

By analyzing the decrease in compressive strength (Table 3) after thermal load, it was noted that with the increase in the *w*/*c* ratio, the degree of material degradation also increased. These results confirm the generally well-known fact that a cement matrix with a higher compressive strength is more resistant to elevated temperatures (also thermal shock).

It is noteworthy that smaller decreases in compressive strength were observed for the CNT samples. This phenomenon is especially visible for the series made of CEM I 52.5R; the *f_c_* decrease for the series C52 was in the range of 29–46%, and for the series C52CNT, 25–30%. This indicates the positive effect of the CNT on the mechanical strength of cement matrix under the influence of elevated temperature, despite the increased porosity of the material. CNTs (well dispersed in a cement matrix) may form bridges between hydrates, which result in an increase in local stiffness of the CSH phase [21,22]. Also, other phenomenon occur, such as crack bridging [52,53,54], delay in micro crack propagation [52], and chemical binding of hydrated phases [23,54,55]. Thus, all the above mentioned mechanisms may result in an increase in the coherence of the cementitious material subjected to thermal shock.

The use of the above described method of the temperature loading caused the formation of thermal cracks due to the volume deformation of sample—in the heating phase, the thermal expansion of the material; in the cooling phase, the shrinkage. The steam pressure created in the pores and capillaries of cement paste causes crack propagation, and transformation of the technological cracks that are present in the structure of the material before applying service loads, into macro-cracks visible on the sample’s surface. In this way, the cement paste structure defects were extracted without excessive deterioration of the mechanical properties of the tested material.

**Figure 15:**
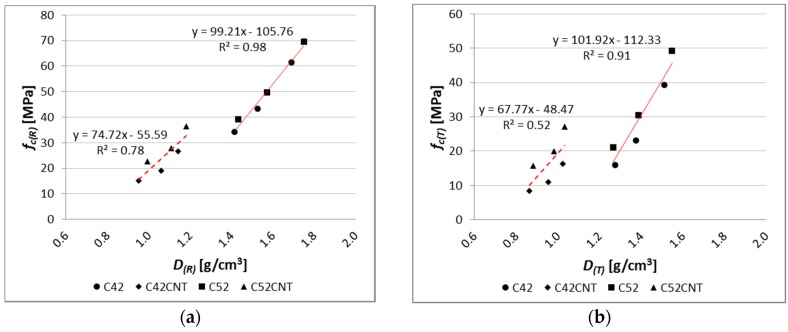
Relationship between compressive strength and apparent density of tested cement pastes for (**a**) reference samples; (**b**) samples after thermal load.

A relationship between compressive strength and apparent density can be observed (Figure 15). This dependence was described by linear regression equations, separately for the classical cement pastes (C42, C52) and the cement matrix containing CNT (C42CNT, C52CNT). The determination coefficient (*R*^2^) of the equation describing the relationship for the classical cement pastes was equal to 0.98 and 0.91, respectively, for the reference and heat-treated samples. It means that the applied model of regression covers more than 90% of achieved data. For the cement pastes with CNT, the *R*^2^ was equal to 0.78 and 0.52. In the case of the reference samples, the good fit of the regression line to the empirical data is observed. The *R*^2^ value for samples after thermal load is not very high, but it should be kept in mind that the equation describes the dependence on materials made of two different cements.

#### 4.2.2. Flexural Strength

As with compressive strength, one of the major factors which shapes bending tensile strength, is the *w*/*c* ratio. Based on the results shown in Figure 9, the cement pastes with *w*/*c* = 0.5 obtained higher *f_ct_*_,*f*(*R*)_ and *f_ct_*_,*f*(*T*)_ values by an average of 26% and 46%, respectively, than samples with *w*/*c* = 0.6. The same relation between samples, both for *w*/*c* = 0.4 and 0.6; 65% and 82%. Clearly, the difference between the results obtained is higher for the heat-treated samples, in terms of *w*/*c*. Cement paste with *w*/*c* = 0.6 contains the most water, which results in the highest vapor pressure during heating. This phenomenon, combined with the thermal deformation of the material, results in a greater decrease in the thermal shock resistance with the higher *w*/*c* ratio.

The differences in *f_ct_*_,*f*(*R*)_ between samples with and without CNTs are less than in the case of *f_c_*_(*R*)_. The bending tensile strength of the reference samples of series C52CNT and C42CNT is reduced by approximately 30%, in comparison to the series C52 and C42. It should be remembered that the CNT cement pastes have much higher porosity than conventional cement matrix, which is the main cause of reduced strength.

On the other hand, after exposure to the elevated temperature, particularly in the case of samples containing CEM I 52.5R, the C52CNT samples had higher *f_ct_*_,*f*(*T*)_ values, by an average of 18%, than series C52. The beneficial effect of using CNTs is larger, the lower the *w*/*c* ratio is. It can be stated, as in the case of *f_c_*, that the CNT forms mechanical connections with the cement hydration products in the material’s nano-structure. This translates into increased cohesion of cement paste in the aspect of its stretching. It is so important that when using the CNT application method, the foam effect was achieved, which translates into a significant reduction in weight of the cementitious material.

**Figure 16:**
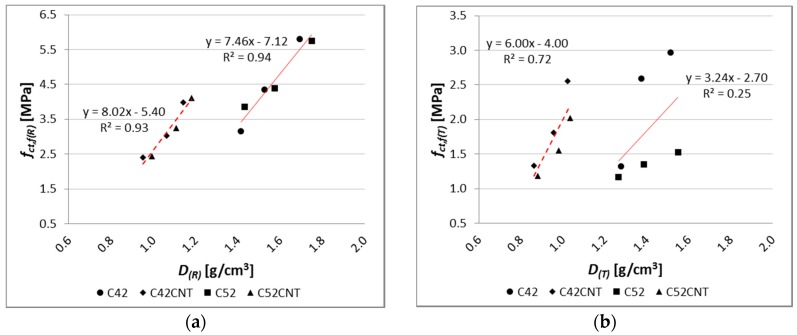
Relationship between bending tensile strength and apparent density of tested materials for (**a**) reference samples; (**b**) samples after thermal load.

There is a very strong relationship between bending tensile strength and apparent density of the modified cement pastes (Figure 16), but only for the reference samples. The coefficient of determination of the linear regression equation reaches a very high value, equal to 0.93 and 0.94, respectively, for pastes with and without CNT. On the other hand, after the influence of the elevated temperature, the regression model no longer reflects empirical data in such a good way (*R*^2^ = 0.72 for the CNT matrix, *R*^2^ = 0.25 for the classical cement paste). In addition, on the *f_ct_*_,*f*_ and *D* graph, the main advantage of the CNT cementitious material is visible—the reduction in weight without loss of bending tensile strength (mainly after the influence of the elevated temperature).

By analyzing the results shown in Figure 9, it can be observed that the class of cement has very little effect on the *f_ct_*_,*f*(*R*)_ results. The cement pastes made of CEM I 52.5R reached higher *f_ct_*_,*f*(*R*)_ values, only by 5%, than the samples with CEM I 42.5R. The effect of the cement’s class is much higher in the case of cementitious materials with aggregate (mortars and concretes), because the resultant mechanical strength of the material is also determined by the interfacial transition zone (ITZ). In the case of presence of aggregate in the material, the strength of ITZ depends to a large extent on the cement’s class [56].

The effect of the cement’s class is clearly visible after the thermal load. Samples made of the cement of higher class (CEM I 52.5R) achieved lower *f_ct_*_,*f*(*T*)_ values by an average of 30% from the CEM I 42.5R specimens. This is due to the material’s fragility. As the compressive strength of cement composite increases, the material’s fragility also increases. Fragility, as a physical property of a material, consists in its cracking under the influence of external forces. Materials that are fragile absorb relatively little energy before cracking. The fragility [1] of cement matrix is a ratio of bending tensile strength (*f_ct_*_,*f*_) to compressive strength (*f_c_*). Then, the fragile material is such, for which the ratio is less than 0.125.

Results in Table 5 confirms that cement pastes with the highest compressive strength are usually the most fragile (*w*/*c* = 0.4). The class of cement has a much greater effect on the fragility than, for example, the *w*/*c* ratio. For example, the fragility of the reference samples made of CEM I 52.5R is higher by an average of 19%, than cement pastes made of CEM I 42.5R. After the temperature load, this difference is over 2.5 times bigger, and is equal to 52%.

The presence of CNTs in the cement matrix has reduced fragility both before and after exposure to the elevated temperature by 45% and 77%, respectively. As the length to width ratio of the CNTs applied is significant (length 1500 times larger than diameter), the CNT anchorage in the hydrates is locally large in length. It is likely that during tensile stress growth in the cementitious material, the CNTs slide in the anchor, which results in the change of fragility of the CNT pastes towards the materials with plastic properties [21,22]. The CNTs’ tensile strength is several tens of thousands of times greater than the cement paste, so it is not possible to break CNTs at the stresses present in cement matrix.

When analyzing the decrease in bending tensile strength (Table 3) after temperature load, no strict relationship between this parameter and the *w*/*c* ratio was observed.

As in the case of *f_c_*, the increased resistance of the CNT pastes to the thermal shock was observed because the decrease of *f_ct_*_,*f*_ was in the range of 36–52%, and for the samples of the classical cement paste, 40–73%. Smaller losses in bending tensile strength showed samples made of CEM I 42.5R, which is indirectly related to the fragility phenomenon described above.

### 4.3. Correlations Between Parameters

Table 6 shows a matrix with the Pearson’s correlation coefficients (*r*) between the investigated parameters of the cement pastes. It is worth noting that almost all parameters are very strongly (*r* > 0.9) or strongly (*r* > 0.7) correlated. A change in the parameters tested results in a closely linked change in the remaining characteristics of the cementitious material. This knowledge is useful for estimation purposes.

Only the dependence of the bending tensile strength measured after the temperature load (*f_ct_*_,*f*(*T*)_) with the other parameters is weakly correlated (*r* < 0.4), and this parameter is not suitable for proper estimation of other properties both before and after the impact of the thermal shock.

### 4.4. Microstructure of the Material

SEM and EDS allowed qualitative and quantitative analysis of a local microstructure of the material. Below, selected images of microstructure of the cement pastes after thermal load are shown and described.

**Figure 17:**
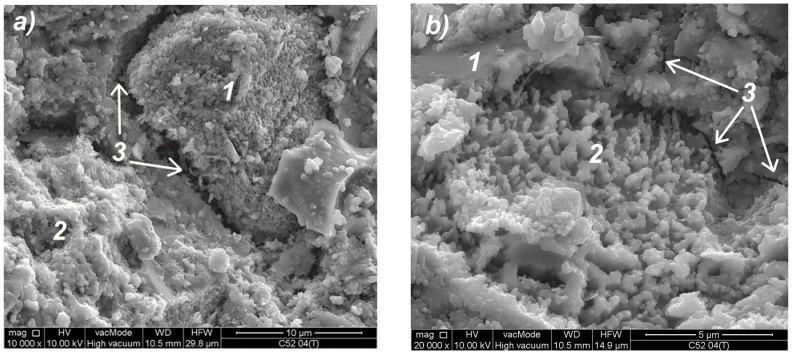
SEM photos of the C52 cement paste microstructure (descriptions of the indications in the text) for (**a**) magnification 10,000×; (**b**) magnification 20,000×.

Figure 17a shows the microstructure of a classic cement paste (C52). The CSH phase is shown in the form of small isometric grains of hydrated calcium silicates (1) and as a congested gel (2), which corresponds to the type III and IV phases of the CSH, according to the classification proposed by Diamond [57]. In the middle of the photo, there is a clear crack (3) with residual bridges formed from the CSH phase. In the upper part of Figure 17b, a portlandite plate (1) can be observed with hydrated calcium silicates. In the middle of the image (2) there is a CSH phase at borderline of II (the honeycomb CSH phase) and III Diamond’s type. A microcrack (3) passing through the CSH gel is also visible.

**Figure 18:**
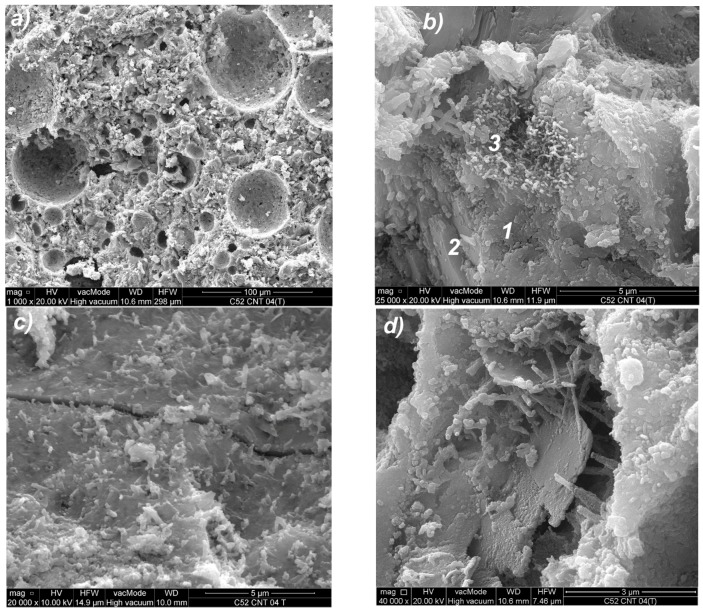
SEM photos of the C52CNT cement paste microstructure (descriptions of the indications in the text) for (**a**) magnification 1000×; (**b**) magnification 25,000×; (**c**) magnification 20,000×; (**d**) magnification 40,000×.

The use of CNTs in the form of an aqueous dispersion with SDS, as mentioned earlier, caused the foaming of the cement matrix. The result of this process is the increased porosity of the material, with the predominant proportion of pores with diameters greater than 50 μm. Most of these pores are oval or spherical, as shown in Figure 18a. Such pore shape is characteristic for the foamed cement matrix.

The microstructure of the CNT paste differs from the classical cement paste, not only in terms of porosity, but also in the morphology and chemistry of the material. Thus, Figure 18b shows the condensed CSH gel mainly type IV, with few monosulfates (1) interspersed with calcium hydroxide crystals (2); the area of higher CNT coverage with surfactant (3) was also noted. This may indicate insufficient dispersion of CNTs in the dispersion medium at the suspension preparation stage. The reason may also be the CNTs’ conglomeration into larger aggregates during mechanical mixing. The micro-cracks, which are present in the microstructure, mostly pass through the condensed CSH gel, as shown in Figure 18c. Figure 18d shows portlandite plates in the void of material that are connected by CNTs and thin ettringite needles.

On the same samples, the EDS X-ray microanalysis was performed, the results of which are shown in Table 4. Cement hydration products are predominantly thermally and chemically stable at the furnace temperature (250 °C). Thus, the resulting chemical composition greatly reflects the material’s composition in a state before being subjected to the temperature load. According to Zhang and Ye [58], from 30 °C to 400 °C, the microstructure of cement paste does not change too much—the volumes of capillary pore from room temperature to 400 °C is almost the same. Other literature reports show that within the temperature range up to 250 °C, the evaporation of free water and the decomposition of some components of cement matrix, such as tobermorite gel [59] and gypsum [60], mainly occurs. Also, a reduction in AFm phases (hydrated calcium aluminates) content was observed [61]. Ettringite is intrinsically unstable in a cement paste above about 70 °C, but if enough sulfate is present, it can exist in cement pastes at temperatures up to at least 90 °C [61,62]. Other cement hydration products are characterized by a high chemical stability in the temperature range analyzed. 

Characteristic for hardened cementitious materials is the high content of SiO_2_ and CaO, as these oxides correspond to the CSH phase (in the 28 day cement paste, the content of the CSH phase can exceed 70–80%), and cement hydration products, such as portlandite. The EDS and SEM analysis confirmed that hydrated calcium silicates present in various morphological forms are the primary constituent of the hardened cement paste.

In the case of the CNT samples, the SiO_2_ content is noticeably lower (17.2% less than the unmodified cement paste). Additionally, the reduction of aluminum and magnesium phases were observed (23.1% and 12.8%, respectively). A weight content reduction of hydrated phases is caused by additional porosity, which is the main reason for the decrease in the material strength. 

Modification of the cement matrix due to the presence of SDS was also detected—the SO_3_ content is greater by 37.4%, compared to the conventional cement paste. It is also interesting to note that the CNT paste is characterized by the higher content of sulfur than aluminum compounds. This is rarely observed when Portland cement is used.

Literature analysis indicates the very high stability of CNTs in water dispersion with addition of SDS, however, the surfactant used adversely modifies the chemistry of cement matrix. For this purpose, alternative surfactants should be sought, which would result in a good degree of CNT dispersion in water, with no reactivity (or reduction of adverse effects to the minimum) with the cement paste components.

Another option to improve performance of the CNT paste may be the application of defoaming agents [23,25,63]. For example, tests were performed using oligomers of polyether [64] and tributyl phosphate [65] as defoaming agents. In both cases, defoamer improved ultrasonic SDS dispersion by breaking thick bundles into thinner pieces, and decreased the amount of air bubbles.

However, further studies on the use of CNT in the presence of SDS, due to the foaming properties of the surfactant, may be the basis for the development of a modified cement binder used in the production of aerated concretes, or in combination with lightweight aggregates, for the production of lightweight concretes.

## 5. Conclusions

CNT is the material that has revolutionized materials engineering, and scientists around the world are exploring the use of CNTs in many areas (e.g., medicine, electronics, materials, etc.). This paper describes the results of investigations concerning the possibility of using CNTs to modify the cement matrix. A number of physical and mechanical parameters were examined, including apparent density, linear shrinkage, bending tensile and compressive strength. The correlations between these parameters were calculated. The microstructure of the modified cement pastes was also analyzed by means of SEM and EDS. Moreover, the behavior of the CNT cementitious material subjected to the short-term thermal stress at 250 °C was investigated, which is the first such test described in the literature.

A thorough analysis of the obtained results enables the formulation of the following conclusions:

1. The use of the aqueous CNT dispersion using the SDS surfactant caused foaming of the cement paste. This is due to the fact that SDS, as an anionic surfactant, has characteristics specific to blowing agents.
2. Due to the foam effect, cement pastes with CNTs were characterized by a lower apparent density, by 31% compared to the conventional cement pastes; the same density difference occurred both before and after the thermal load.
3. Samples containing CNTs were characterized by a large initial growth rate of shrinkage (after 3 days of maturing, the shrinkage was as much as 140% greater than the unmodified samples); at the time of maturing, the increase in shrinkage significantly decreased.
4. After the temperature load, the change in linear shrinkage of samples with and without CNT was similar, and ranged from 135–174% with respect to the reference values.
5. A very high dependence between shrinkage and apparent density of modified cement pastes was observed (correlation coefficients ranged from −0.95 to −0.98).
6. CNT modified cement pastes have an average of 50% less compressive strength compared to the conventional cement matrix, due to the increased porosity.
7. The CNT addition increased the resistance of the cement matrix to elevated temperatures in terms of the compressive strength; in the case of CEM I 52.5R, the reduction in the compressive strength of CNT modified samples ranged between 25–30%, and for unmodified samples, 29–46%.
8. Bending tensile strength of the pastes with CNT (before thermal load) was about 30% lower than the pastes without the additive.
9. After exposure to the elevated temperature, in the samples containing CEM I 52.5R, CNT modified cement pastes achieved about 18% greater bending tensile strength compared to the unmodified samples. It has been found that the CNT additive forms mechanical connections with the cement hydration products in the material’s nano-structure, and improves the cohesion of the cement paste, both in terms of its stretching and compression. This is important because of the CNT application method used, where a foam effect was achieved—a significant reduction in the weight of the cementitious material.
10. Increased thermal resistance of the cement pastes with CNTs in the aspect of the bending tensile strength of cement matrix was observed; the reduction of this parameter for the CNT samples ranged between 36–52%, and for the samples of the classical cement paste, 40–73%.
11. SEM and EDS analysis allowed for the identification of structural differences between individual samples of the cementitious material. It has been noted that the presence of CNTs with SDS adversely affects the chemistry of the cement paste (e.g., lower content of SiO_2_, greater content of CaO, SO_3_).
12. Further studies on the use of CNTs in conjunction with SDS, for the modification of cement matrix, may contribute to the development of a modified cement binder used in the manufacture of lightweight or aerated concrete.

## Figures and Tables

**Table 1:** Chemical and mineral components, and the Blaine specific surface area of the Portland cements used.

Cement’s Class	Chemical Analysis (%)								
	SiO_2_	Fe_2_O_3_	Al_2_O_3_	CaO	MgO	SO_3_	Cl	Na_2_O	K_2_O
CEM I 42.5R	20.18	3.39	4.38	64.79	1.17	2.91	0.083	0.26	0.49
CEM I 52.5R	20.19	3.30	4.33	64.76	1.17	3.16	0.078	0.26	0.48
**Mineral Composition (%)**					**Blaine Specific Surface Area (cm^2^/g)**				
**Cement’s Class**	**C3S**	**C2S**	**C3A**	**C4AF**					
CEM I 42.5R	63.41	8.92	5.88	10.31	4010				
CEM I 52.5R	62.97	9.28	5.90	10.03	4596				

**Table 2:** The linear shrinkage values of samples tested after thermal load, the percentage increase in shrinkage, and apparent density decrease due to the thermal shock.

Series	C42			C42CNT			C52			C52CNT		
*w*/*c*	0.4	0.5	0.6	0.4	0.5	0.6	0.4	0.5	0.6	0.4	0.5	0.6
*S*_28(*T*)_ (mm/m)	5.50	6.35	7.58	8.60	10.15	10.92	4.53	5.90	7.48	8.67	10.10	11.06
Δ*S=S*_28(*T*)_ − *S*_28(*W*)_ (mm/m)	3.25	3.83	4.35	5.04	6.38	6.86	2.84	3.46	4.35	5.50	6.35	6.67
Increase of *S*_28_ (%)	144.4	152.1	134.8	141.5	169.1	168.8	168.5	141.9	139.3	173.7	169.4	151.7
Decrease of *D* (%)	10.6	9.9	9.8	10.3	10.0	9.2	11.5	11.8	11.6	12.4	11.5	11.3

**Table 3:** Percentage decrease in the compressive and bending tensile strength due to the thermal shock.

Series		C42			C42CNT			C52			C52CNT		
*w*/*c*		0.4	0.5	0.6	0.4	0.5	0.6	0.4	0.5	0.6	0.4	0.5	0.6
Decrease of	*f_c_* (%)	36.1	46.9	53.5	39.0	42.5	45.1	29.1	38.8	46.0	25.4	28.5	30.3
	*f_ct_*_,*f*_ (%)	48.8	40.4	58.2	35.8	40.2	44.9	73.4	69.2	69.9	50.9	52.2	51.7

**Table 4:** Oxide composition of cement paste after the elevated temperature interaction, and percentage difference in a content of individual components with respect to series C52.

Series	Oxide Composition (% by Weight)							
	Na_2_O	MgO	Al_2_O_5_	SiO_2_	SO_3_	K_2_O	CaO	Fe_2_O_3_
C52	0.79	1.06	6.58	22.87	3.78	0.52	61.30	3.12
C52CNT	0.58	0.92	5.06	18.93	5.20	0.94	65.02	3.34
Difference between series (%)	−26.0	−12.8	−23.1	−17.2	+37.4	+80.9	+6.1	+6.8

**Table 5:** The fragility of the modified cement pastes expressed in *f_ct_*_,*f*_/*f_c_* ratio.

Series	C42			C42CNT			C52			C52CNT		
*w*/*c*	0.4	0.5	0.6	0.4	0.5	0.6	0.4	0.5	0.6	0.4	0.5	0.6
*f_ct_*_,*f*(*R*)_/*f_c_*_(*R*)_	0.094	0.100	0.092	0.149	0.159	0.158	0.083	0.088	0.099	0.113	0.117	0.108
*f_ct_*_,*f*(*T*)_/*f_c_*_(*T*)_	0.076	0.112	0.083	0.157	0.165	0.159	0.031	0.044	0.055	0.074	0.078	0.075

**Table 6:** Correlation matrix of the parameters examined of the cement pastes.

Parameters	*f_c_*_(*R*)_	*f_c_*_(*T*)_	*f_ct_*_,*f*(*R*)_	*f_ct_*_,*f*(*T*)_	*D*_(*R*)_	*D*_(*T*)_	*S*_28(*R*)_	*S*_28(*T*)_	Δ*S*
***f_c_*_(*R*)_**	1.00	-	-	-	-	-	-	-	-
***f_c_*_(*R*)_**	0.96	1.00	-	-	-	-	-	-	-
***f_ct_*_,*f*(*R*)_**	0.94	0.92	1.00	-	-	-	-	-	-
***f_ct_*_,*f*(*T*)_**	0.29	0.26	0.54	1.00	-	-	-	-	-
***D*_(*R*)_**	0.95	0.84	0.87	0.27	1.00	-	-	-	-
***D*_(*T*)_**	0.94	0.82	0.87	0.29	1.00	1.00	-	-	-
***S*_28(*R*)_**	−0.96	−0.89	−0.93	−0.34	−0.96	−0.95	1.00	-	-
***S*_28(*T*)_**	−0.95	−0.84	−0.91	−0.33	−0.98	−0.98	0.98	1.00	-
**Δ*S***	−0.92	−0.80	−0.88	−0.32	−0.98	−0.98	0.95	0.99	1.00
